# Clinical Profile of Acute Kidney Injury in Type 2 Diabetes Mellitus Adult Patients Presenting With Diabetic Ketoacidosis: A Cross-Sectional Study

**DOI:** 10.7759/cureus.80183

**Published:** 2025-03-06

**Authors:** Sajit Varghese, Anna Mary Thomas, Arjun V, Acsa J Solamon

**Affiliations:** 1 Department of General Medicine, Pushpagiri Institute of Medical Sciences & Research Centre, Thiruvalla, IND

**Keywords:** acute kidney injury (aki), diabetic ketoacidosis (dka), icu length of stay (iculs), renal function assessment, type 2 diabetes mellitus

## Abstract

Objective: The purpose of this cross-sectional study was to identify the characteristics of acute kidney injury (AKI) in adult patients with type 2 diabetes mellitus (T2DM) who were admitted with diabetic ketoacidosis (DKA).

Methodology: One hundred patients were selected based on the inclusion and exclusion criteria. Kidney function was assessed using kidney estimated glomerular filtration rate (KeGFR) calculations based on the Kidney Disease: Improving Global Outcomes (KDIGO) criteria. Data collection included clinical, biological, and demographic information. Univariate and multivariate logistic regression analyses were performed, with a p-value of less than 0.05 considered statistically significant. The study also examined risk factors, intensive care unit (ICU) treatments, and clinical outcomes.

Results: AKI was significantly associated with ICU length of stay (p = 0.002), and all patients with prolonged ICU admission developed this condition. A higher incidence of AKI was observed in patients requiring mechanical ventilation, renal replacement therapy, or inotropic support. Among the 100 patients included in the study, 94 achieved clinical recovery, including 76 who had AKI, while six patients did not survive.

Conclusion: AKI is a common complication of DKA in adults with T2DM, particularly in hot climate regions. Most cases were detected within the first 24 hours, classified as stage 1 severity, and were pre-renal. Early detection plays a crucial role in preventing complications and improving patient recovery. KeGFR calculations proved an effective tool for monitoring kidney function changes in these patients.

## Introduction

Globally, type 2 diabetes mellitus (T2DM) is among the most prevalent health concerns. It occurs when the body develops insulin resistance, leading to elevated blood sugar levels and a partial lack of insulin, in contrast to the complete insulin deficiency in type 1 diabetes mellitus (T1DM) [1]. One serious but treatable complication of T2DM is diabetic ketoacidosis (DKA), which arises due to uncontrolled hyperglycemia, severe dehydration, reduced insulin activity, and an increase in counter regulatory hormones such as cortisol, glucagon, catecholamines, and growth hormone [2].

DKA is a life-threatening condition characterized by hyperglycemia, metabolic acidosis, and ketonemia [3]. It carries a high risk of recurrent morbidity and mortality. The rising global prevalence of T2DM among adults has been linked to an increasing incidence of acute DKA, commonly triggered by poor treatment adherence, concurrent infections or sepsis, alcohol or drug reactions, and cardiovascular or cerebrovascular events [4].

Acute kidney injury (AKI) is defined as a sudden decline in kidney function (within hours) and is often triggered by sepsis, ischemia, toxicity, or metabolic imbalances that impair renal function [5]. DKA leads to severe hypovolemia, tubular injury, and multiple metabolic disturbances, making AKI a common complication in at least 50% of hospitalized DKA patients. The presence of AKI in DKA prolongs hospital stays, increases mortality risk, and raises susceptibility to chronic kidney disease (CKD) [6].

Most of the available scientific literature on AKI in DKA patients focuses on renal impairment in T1DM patients, particularly within the pediatric population. However, the characteristics and risk factors of DKA in T2DM patients remain less explored, as does the incidence of AKI in this population [7]. This study seeks to address the gap in knowledge regarding the incidence and characteristics of AKI in adults with T2DM who develop DKA. While existing research predominantly focuses on DKA and AKI in T1DM, particularly in pediatric populations, there is limited understanding of these complications in adults with T2DM. By examining this group, the study aims to identify key risk factors and clinical characteristics associated with AKI in the context of DKA, thereby contributing to improved management strategies and better outcomes for patients with T2DM and DKA. The primary objective is to estimate the incidence of AKI in adult patients with T2DM admitted with DKA to a tertiary care hospital and to identify the clinical characteristics of AKI in these patients.

## Materials and methods

This study was conducted at Pushpagiri Institute of Medical Sciences & Research Centre (PIMSRC), a tertiary care medical college hospital in South India, as a cross-sectional investigation at a single center. The study commenced on December 1, 2023, and continued until December 31, 2024, following approval from the institutional review board of PIMSRC (IRB/03/10/2023) on November 15, 2023.

Study participants

The study population consisted of adult patients with T2DM who were admitted with a diagnosis of DKA under the Department of General Medicine, PIMSRC, Thiruvalla, Kerala, India, during the study period. Informed consent was obtained from all participants or their authorized representatives before inclusion.

Inclusion and exclusion criteria

Patients were included in the study if they were 18 years or older and had a confirmed diagnosis of T2DM with DKA at the time of admission. Patients younger than 18 years, those with a known diagnosis of T1DM, or those who were pregnant were excluded. Additional exclusion criteria included patients undergoing chemotherapy, those diagnosed with human immunodeficiency virus (HIV), terminally ill patients, and individuals with known malignancies.

The sample size for this cross-sectional study was determined based on previous research by Orban et al. [8]. The calculation was performed using the standard normal ordinate (Z = 1.96) for a 95% confidence level, an expected prevalence (P = 0.5), a relative precision (D = 0.1), and a 10% margin of error. Using these parameters, 100 patients were selected through a consecutive sampling technique.

Operational definitions and study variables

DKA was diagnosed based on three key indicators: blood glucose > 250 mg/dL, metabolic acidosis (pH < 7.3), and ketones in blood or urine. AKI was identified according to the KDIGO criteria, defined as an increase in serum creatinine by at least 0.3 mg/dL within 48 hours, a 50% rise in serum creatinine from baseline within one week, or urine output < 0.5 mL/kg/hour for at least six hours. AKI severity was classified into three stages: stage 1 included reduced urine output for six to 12 hours, stage 2 involved decreased urine output for more than 12 hours, and stage 3 was marked by very low or no urine output for at least 24 hours.

AKI was further classified based on its underlying cause. Pre-renal AKI resulted from reduced kidney perfusion, renal AKI was caused by direct kidney injury or inflammation, and post-renal AKI was associated with urinary tract obstruction leading to congestion and backflow. Diagnosis was established through clinical history, physical examination, urine analysis, and ultrasound imaging. Kidney function was assessed using the kinetic estimated glomerular filtration rate (KeGFR) calculator, providing real-time evaluation of renal function. Estimated glomerular filtration rate (eGFR) values < 45 mL/min/1.73 m² were categorized as very low, while values ≥ 45 mL/min/1.73 m² were classified as low.

Demographic and clinical variables

Demographic data, including age, gender, and body mass index (BMI), were recorded. Patients were categorized into two age groups: group A (18-45 years) and group B (>45 years). BMI was used to classify patients as obese (BMI ≥ 30 kg/m²) or non-obese (BMI < 30 kg/m²). These classifications were used to analyze the impact of age and body weight on AKI occurrence and severity in DKA patients.

Comorbidities and vital signs

Comorbid conditions, including hypertension (HTN), coronary artery disease (CAD), obstructive airway disease (OAD), CKD, and chronic liver disease (CLD), were documented. Vital parameters recorded at admission included pulse rate and systolic blood pressure (SBP). Pulse rate was classified as tachycardia (≥100 bpm), bradycardia (<60 bpm), or standard (60-100 bpm). SBP was categorized into normotensive (120-140 mmHg), hypertensive (>140 mmHg), or hypotensive (<120 mmHg).

Biochemical and laboratory assessments

Serum creatinine was measured using an automated biochemical analyzer (Beckman Coulter TBA 120FR). Arterial blood gas (ABG) analysis recorded pH, bicarbonate (HCO₃⁻), and arterial oxygen pressure. Glycated hemoglobin (HbA1C) levels were measured using the Bio-Rad D-10 high-performance liquid chromatography (BIORAD D10 HPLC) machine, and blood glucose levels were assessed using the glucose oxidase-peroxide (GOD-POD) method. Additional laboratory parameters recorded included serum albumin, serum calcium, serum uric acid, total white blood cell count, hemoglobin levels, and platelet counts.

Clinical course and outcomes

The clinical course of the patients was assessed using ICU length of stay (ICULS), which was categorized as short if it ranged between zero and two days, medium if it ranged between three and five days, and long if it was ≥6 days. The study also recorded the need for inotropic support, renal replacement therapy, and mechanical ventilation. Clinical recovery was considered adequate if the patient was discharged in a hemodynamically stable state with improving AKI. It was deemed inadequate if the patient expired, was referred, or was discharged against medical advice.

Data analysis

Test results, physical examinations, and a comprehensive review of the patient's medical history were used to compile the information acquired on the patient. The IBM SPSS Statistics for Windows, Version 25.0 (released 2017, IBM Corp., Armonk, NY), was utilized for statistical analysis. The mean values and standard deviations were employed in the continuous data analysis, while the frequencies and percentages were used to classify categorical data. The sample was considered statistically significant when the p-value was less than 0.05. Using univariate and multivariate analysis, a comparison was made between patients with acute renal injury and those who did not have such an injury.

## Results

Table 1 compares demographics and comorbidities in DKA patients with and without AKI. The mean age distribution was similar between the two groups, with no statistically significant difference (p = 0.087). A significantly higher proportion of obese patients developed AKI compared to non-obese patients (p = 0.044). The sex distribution did not show a significant difference (p = 0.63). Among comorbidities, CKD and CAD were significantly associated with AKI in DKA patients (p = 0.02 and p = 0.036, respectively). HTN, OAD, and CLD did not show statistically significant differences between the AKI and non-AKI groups (p > 0.05). These findings suggest that obesity, CKD, and CAD may contribute to an increased risk of AKI in DKA patients, highlighting the importance of monitoring these factors in clinical practice.

**Table 1:** Comparison of demographics and comorbidities in DKA patients with and without AKI * shows significant p-value. DKA: diabetic ketoacidosis, AKI: acute kidney injury

Demographics		Total (N = 100)	AKI (N = 82)	Non-AKI (N = 18)	p-value (significant <0.05)
Age	18-45 years	43	32	11	0.087
	>45 years	57	50	7	
BMI	Non-obese	69	53	16	0.044
	Obese	31	29	2	
Sex	Female	44	37	7	0.63
	Male	56	45	11	
Comorbidities					
HTN (hypertension)	No	25	17	8	0.067
	Yes	75	65	10	
CAD (coronary artery disease)	No	74	57	17	0.036*
	Yes	26	25	1	
OAD (obstructive airway disease)	No	74	58	16	0.144
	Yes	26	24	2	
CKD (chronic kidney disease)	No	80	62	18	0.02*
	Yes	20	20	0	
CLD (chronic liver disease)	No	91	73	18	0.357
	Yes	9	9	0	

**Figure 1:**
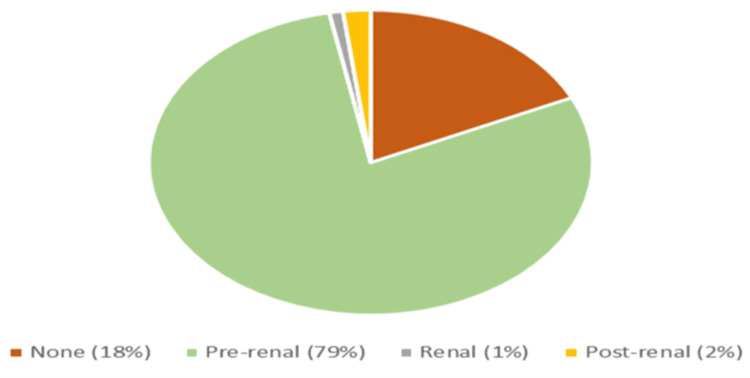
Distribution of acute kidney injury (AKI) based on clinical type Image created by the authors using Microsoft Excel 2021

**Figure 2:**
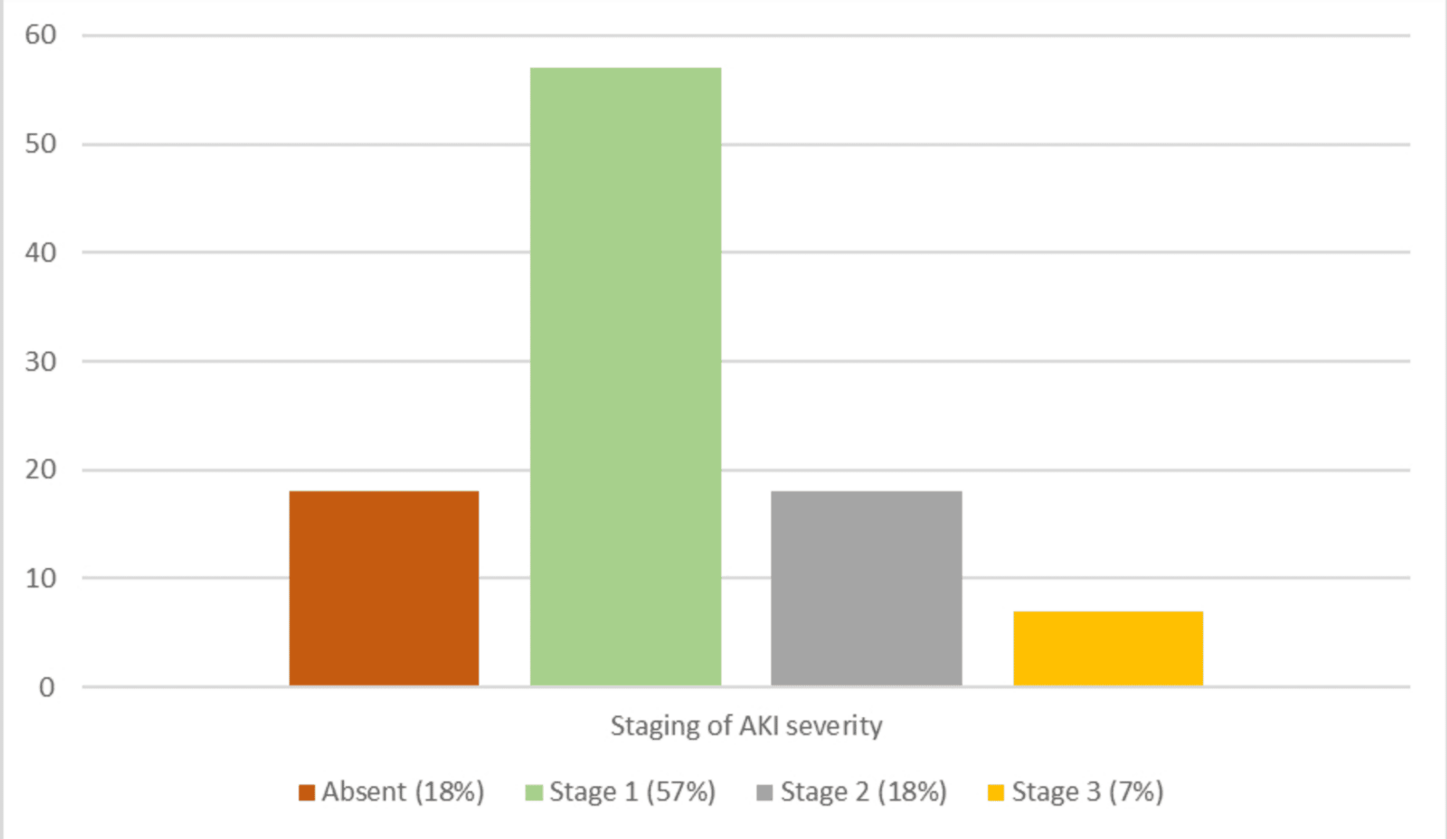
Distribution of acute kidney injury (AKI) severity staging Image created by the authors using Microsoft Excel 2021

In 79 out of 82 patients, AKI developed within the first 24 hours, with KeGFR classified as very low in 22 patients and low in 60 patients (Figures 1, 2).

Bradycardia, tachycardia, abnormal SBP, metabolic acidosis, hypoxemia, high HbA1C, and abnormal biochemical markers were significantly correlated with AKI, except for platelet counts (Table 2).

**Table 2:** Comparison of clinical characteristics in patients with and without acute kidney injury (AKI) * shows significant p-value

Clinical characteristics		Total (n = 100)	AKI (n = 82)	Non-AKI (n = 18)	p-value significant at p < 0.05
Pulse rate on admission	Bradycardia	8	8	0	0.024*
	Normal	5	2	3	
	Tachycardia	87	72	15	
Systolic blood pressure	Hypotensive	37	36	1	0.000*
	Normotensive	12	3	9	
	Hypertensive	51	43	8	
Arterial pH	Very low	22	22	0	0.010*
	Low	78	60	18	
Bicarbonate	Very low	22	22	0	0.010*
	Low	78	60	18	
Hypoxemia	Absent	66	49	17	0.005
	Present	34	33	1	
HbA1C	Controlled	23	6	17	0.000*
	Uncontrolled	77	76	1	
Blood glucose	High	66	48	18	0.001*
	Very high	34	34	0	
Serum albumin	Low	48	48	0	0.000*
	Normal	52	34	18	
Serum calcium	Low	35	35	0	0.001*
	Normal	65	47	18	
Serum uric acid	High	25	25	0	0.005
	Normal	75	57	18	
Total leukocyte count	Leukopenia	9	9	0	0.002*
	Normal	13	6	7	
	Leukocytosis	78	67	11	
Haemoglobin	Low	37	36	1	0.002*
	Normal	63	46	17	
Platelet counts	Low	14	14	0	0.068
	Normal	86	68	18	

**Figure 3:**
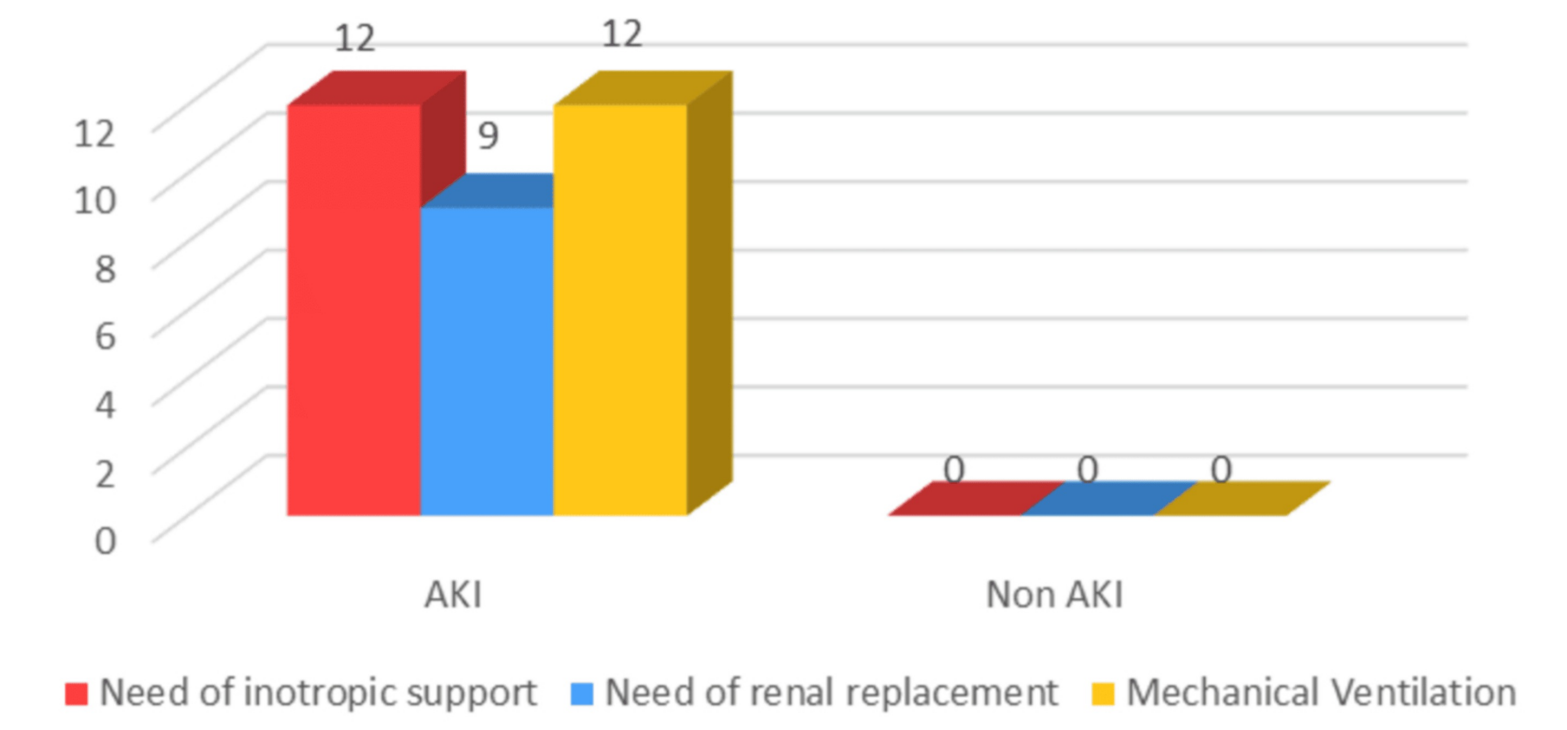
Comparison of clinical management in patients with and without acute kidney injury (AKI) Image created by the authors using Microsoft Excel 2021

Figure 3 illustrates the clinical management interventions required in DKA patients with and without AKI. Twelve AKI patients required inotropic support, nine required renal replacement therapy, and 12 required mechanical ventilation. By contrast, none of the non-AKI patients required these interventions. ICULS was significantly associated with AKI (p = 0.002), with all patients requiring prolonged ICU admission developing AKI. These findings indicate that AKI in DKA patients is associated with a significantly higher need for intensive management, including hemodynamic support, renal replacement therapy, and mechanical ventilation, highlighting the severity of AKI in DKA and underscoring the importance of early identification and intervention.

Clinical recovery was observed in 94 patients, including 76 with AKI, while six patients died. Stage 2 and stage 3 AKI were compared to assess severity (Table 3).

**Table 3:** Comparison of clinical parameters individually with AKI severity staging AKI: acute kidney injury, SBP: systolic blood pressure, KeGFR: kidney estimated glomerular filtration rate

Clinical parameters	AKI	
	Stage 2 with n = 18 (% given in bracket)	Stage 3 with n = 7 (% given in bracket)
Hypotensive SBP	6 (33.33)	6 (85.71)
Very low KeGFR	14 (77.78)	7 (100.00)
Very low arterial pH	14 (77.78)	6 (85.71)
Very low bicarbonate	14 (77.78)	6 (85.71)
Hypoxemia	12(66.67)	6 (85.71)
Uncontrolled HbA1C	17 (94.44)	7 (100.00)
Low serum albumin	17 (94.44)	7 (100.00)
Low serum calcium	15 (83.33)	6 (85.71)
High serum uric acid	10 (55.56)	7 (100.00)
Leukocytosis	15 (83.33)	5 (71.43)
Low hemoglobin	16 (88.89)	7 (100.00)
Low platelet count	4 (22.22)	5 (71.43)

Univariate regression analysis identified hypoxemia, uncontrolled HbA1C, and anemia as strong predictors of AKI, but multivariate analysis confirmed only uncontrolled HbA1C as a statistically significant factor (Table 4).

**Table 4:** Logistic regression analysis * shows significant p-value

Parameters	Univariate		Multivariate	
	Odds ratio (95% CI)	p-value	Odds ratio (95% CI)	p-value
BMI	0.228 (0.049, 1.064)	0.060	-	-
CAD	0.134 (0.017, 1.064)	0.057	-	-
CKD	0.000 (0.000, 0.000)	0.998	-	-
Pulse rate	0.838 (0.315, 2.227)	0.723	-	-
Systolic blood pressure	0.689 (0.384, 1.240)	0.214	-	-
Arterial pH	0.000 (0.000, 0.000)	0.998	-	-
Bicarbonate	0.000 (0.000, 0.000)	0.998	-	-
Hypoxemia	0.087 (0.011, 0.688)	0.021*	0.207 (0.013, 3.188)	0.259
HBA1C	0.005 (0.001, 0.041)	0.000*	0.007 (0.001, 0.064)	0.007
Blood glucose	0.000 (0.000, 0.00)	0.998	-	-
Total leukocyte count	1.254 (0.589, 2.670)	0.558	-	-
Hemoglobin	13.304 (1.690, 104.74)	0.014*	4.971 (0.332, 74.360)	0.245

## Discussion

The incidence of AKI in T2DM patients with DKA is not well established due to a lack of studies in adults. However, available research estimates that it occurs in approximately 50-80% of hospitalized patients [9]. The primary objective of this study was to estimate the incidence of AKI in adult patients with T2DM admitted with DKA. Our study found an incidence of 82%, which may be attributable to the region's tropical, hot, and humid climate, ethnic predisposition, and the socioeconomic composition of the patients included. The average age of the participants was approximately 55 years, with more than half being over 45 years old. Research from 2006 indicated that age plays a significant role in the development of AKI in both males and females, particularly in ICU patients [10]. In line with this, the present study found no significant difference in AKI occurrence between male and female patients.

Malleshappa and Shah [11] concluded that patients with CAD and CKD are at a higher risk of developing AKI. Our study also showed that AKI occurred in a significantly higher proportion of patients with CAD, as well as those with CKD. Thongprayoon et al. [12] discussed various comorbidities associated with the incidence of AKI, including hypertension, chronic liver disease, and obstructive airway disease. However, our study did not reveal any significant association with these comorbidities, although the incidence of acute kidney injury was numerically higher.

Our results revealed that most cases of AKI developed within the first 24 hours of admission, emphasizing the importance of early detection and timely management in DKA patients. In addition, a KeGFR below 45 ml/min/1.73 m² was observed in all seven patients with stage 3 AKI and 14 out of 18 with stage 2 AKI. Thus, KeGFR has been reaffirmed as a reliable estimate of kidney function when creatinine levels are acutely changing, as demonstrated in recent studies [13]. In our study, 57 out of the 82 AKI patients had severity classified as Stage 1, reinforcing that early interventions to correct volume deficits and AKI can help prevent significant kidney damage in these diabetic emergencies. In addition, 79 out of the 82 AKI patients had the pre-renal type, aligning with the predominant mechanisms of AKI in DKA, namely, hypovolemia [14].

Our study findings revealed a significant association between AKI and bradycardia and tachycardia at admission. Bradycardia was observed in eight AKI patients (9.76%), while no cases were reported in the non-AKI group (p = 0.024). By contrast, tachycardia was present in 87% of all patients, occurring in 72 AKI patients (87.8%) and 15 non-AKI patients (83.3%). These results align with previous studies that have linked bradycardia to a higher incidence of AKI [15] and the well-established connection between tachycardia and AKI [16]. In addition, our results showed that patients presenting with either hypotension or hypertension had a significantly higher incidence of AKI compared to those with normal SBP. This underscores the fact that both hypertension and hypotension play a crucial role in the development of AKI, emphasizing the need for careful hemodynamic monitoring and early intervention in patients at risk [17].

Our study findings showed that all 22 patients with severely low arterial pH (<7.20) and bicarbonate levels (<12 mEq/L) developed AKI and experienced a prolonged ICULS. This finding is consistent with Zhu et al. [18], who reported that worsening metabolic acidosis increases the risk of AKI. In addition, our results revealed that 33 out of 34 patients with hypoxemia had AKI, and this association was further confirmed through univariate logistic analysis. These findings align with recent research highlighting the role of hypoxemia and hypoxia-inducible factors (HIFs) in the development of AKI [19], reinforcing the need for early identification and management of metabolic acidosis and hypoxemia to prevent kidney complications.

In the present study, we found that uncontrolled HbA1C (more than 7.5%) and very high blood glucose levels on admission (≥600 mg/dL) were associated with an almost 100% incidence of AKI. In the univariate analysis, HbA1C showed a strong relationship with AKI, and even after adjusting for other factors in the multivariate analysis, uncontrolled HbA1C remained strongly associated with the incidence of AKI. The connection between high blood glucose, HbA1C levels, and an increased risk of AKI has been well-established in recent studies [20].

Hypoalbuminemia is a significant predictor of acute kidney injury, and our study supports this, as all 48 patients with hypoalbuminemia had AKI [21]. Moreover, all seven patients with Stage 3 AKI had hypoalbuminemia, and 17 out of the 18 patients with Stage 2 AKI also had hypoalbuminemia. Although hypocalcemia was significantly present in AKI patients in our study, this was likely due to underlying CKD or CLD in almost all of these patients. In addition, all 25 patients with hyperuricemia had AKI, suggesting a possible role of hyperuricemia in the development of AKI, which warrants further large-scale studies [22].

White blood cell (WBC) count and hemoglobin levels are potential indicators of AKI risk, as demonstrated in our study. We found that all nine patients with leukopenia and 67 out of 78 patients with leukocytosis developed AKI, and this association was statistically significant. These findings align with the study by Seung et al. [23], who reported a U-shaped relationship between WBC count and AKI. In addition, previous research has suggested that anemia increases the risk of AKI, and our results further confirm this association [24]. Anemia (hemoglobin <10.5 g/dL) was observed in 37 patients, and AKI occurred in 36 of them (p = 0.002). Univariate analysis further strengthened this association between low hemoglobin levels and AKI. However, this significance was not observed in multivariate analysis. Some studies have explored the relationship between thrombocytopenia and AKI, but our study did not reveal a statistically significant association [25].

Regarding ICULS, we observed that all patients with medium (three to five days) and long (≥6 days) ICU stays developed AKI. By contrast, in patients who were shifted out of ICU within two days, AKI was 72.7% (48 out of 66 patients). This statistically significant finding indicates that ICULS is directly proportional to AKI incidence, as documented in the literature [26]. Patients with higher-stage AKI (Stage 2 or 3) required critical care interventions such as inotropic support, renal replacement therapy, or mechanical ventilation, highlighting the severity of AKI in these diabetic emergencies.

Limitations

This study is cross-sectional; thus, long-term follow-up was not possible to further validate the associations observed in AKI characteristics. Moreover, only 100 patients were included, and data collection was limited to a single hospital center. Therefore, prognostic stratification and the generalizability of our results require confirmation through larger-scale research.

## Conclusions

AKI is a common complication of DKA in adult type 2 diabetic patients, particularly in hot climate regions. Most cases of AKI in DKA occur early (within the first 24 hours), are pre-renal, and are of Stage 1 severity. Therefore, early detection prevents morbidity and mortality in DKA patients. KeGFR calculation appears to be a reliable measure of acutely changing kidney function in DKA patients. Several factors have been significantly associated with the occurrence of AKI, including obesity, CAD, CKD, abnormal pulse rate, hypotension, severe metabolic acidosis, hypoxemia, uncontrolled HbA1C, very high blood glucose levels, hypoalbuminemia, hyperuricemia, hypocalcemia, abnormal leukocyte counts, and anemia.
